# Supplementary material for: MUSICiAn: genome-wide identification of genes involved in DNA repair via control-free mutational spectra analysis
Source: NAR Genom Bioinform. 2026 Jan 8;8(1):lqaf202. doi: 10.1093/nargab/lqaf202 (PMC12783044; doi:10.1093/nargab/lqaf202)
Supplement: lqaf202_Supplemental_File [file lqaf202_supplemental_file.pdf]

MUSICiAn: Genome-wide Identification of Genes Involved in DNA Repair via Control-Free Mutational Spectra Analysis - Supplementary Materials

Colm Seale[1, 2], Marco Barazas[3], Robin van Schendel[3], Marcel Tijstermann[3], and Joana P. Gonçalves[1]

[1]Pattern Recognition & Bioinformatics, Department of Intelligent Systems, Faculty EEMCS, Delft University of Technology, The Netherlands, [2]Holland Proton Therapy Center, Delft, The Netherlands, and [3]Human Genetics Department, Leiden University Medical Centre, Leiden, The Netherlands

1 Supplementary Tables

Target	Sample	**Before**	Step *i*	Step *ii*	Step *iii*	**Step *iv***
T1	MB01	**89414**	75218	74654	64700	**60975**
	MB02	**89423**	76352	75789	67200	**63347**
T2	MB03	**89492**	80058	79887	79606	**79401**
	MB04	**89481**	82096	81926	80934	**80646**
T3	MB05	**89477**	79296	79042	78796	**78675**
	MB06	**89478**	78492	78237	78112	**78037**

Table S1: Breakdown of sgRNA counts after each QA filtering step as described in "Quality analysis and sgRNA filtering" from the main article. Briefly, we filtered out sgRNAs per replicate by applying the following criteria in order: *(i)* a total mutated read count below 700; *(ii)* only two sgRNA representations for the gene; *(iii)* a median pairwise Pearson correlation coefficient below 0.6 compared to other sgRNAs for the same gene within the same replicate; or *(iv)* a median pairwise Pearson correlation below 0.6 compared to paired replicates at the same target site.

Target	Sample	**Before**	Step *i*	Step *ii*	Step *iii*	**Step *iv***
T1	MB01	**18406**	18023	17887	17130	**17004**
	MB02	**18405**	18025	17889	17251	**17135**
T2	MB03	**18406**	18167	18116	18090	**18089**
	MB04	**18406**	18264	18213	18144	**18140**
T3	MB05	**18406**	18224	18149	18134	**18133**
	MB06	**18406**	18178	18103	18095	**18092**

Table S2: Breakdown of gene counts after each QA filtering step per the steps outlined in Table S1 above.

MUSICiAn Category	SIQ Categories	Description
Wild-type	WT	No mutation.
Deletion with insertion	DELINS<br>TINS<br><br><br>TANDEMDUPLICATION<br><br><br>TANDEMDUPLICATION-_COMPOUND	Deletion with insertion.<br>Deletion with an insertion where the insert is copied from the flank.<br>Duplication of sequence immediately flanking the cut-site.<br>A tandem duplication with some additional inserted sequence.
Insertion	INSERTION	Any simple insertion event.
Homology-directed repair	HDR	Homology-directed repair event.
Deletion with no microhomology<br>Deletion with 1bp microhomology<br>Deletion with 2bp microhomology<br>Deletion with 3+bp microhomology	DELETION	Any simple deletion event. Additional details recorded include the presence and length of any homology between one side of the deleted sequence and the opposing flank of the cut site.

Table S3: Mapping of MUSICiAn mutation categories to SIQ mutation types and details.
